# Association of Social Determinants of Health With Rapid Response Events: A Retrospective Cohort Trial in a Large Pediatric Academic Hospital System

**DOI:** 10.3389/fped.2022.853691

**Published:** 2022-04-21

**Authors:** Nikki R. Lawson, Darlene Acorda, Danielle Guffey, Julie Bracken, Aarti Bavare, Paul Checchia, Natasha S. Afonso

**Affiliations:** ^1^Department of Pediatrics, Baylor College of Medicine, Houston, TX, United States; ^2^Texas Children's Hospital, Houston, TX, United States; ^3^Institute for Clinical and Translational Research, Baylor College of Medicine, Houston, TX, United States; ^4^Department of Pediatrics, Section of Critical Care Medicine, Baylor College of Medicine, Houston, TX, United States

**Keywords:** health care disparities, pediatric rapid response teams, social determinants of health (SDH), language barriers, pediatrics

## Abstract

**Background:**

Social determinants of health (SDH) are known to impact hospital and intensive care unit (ICU) outcomes. Little is known about the association between SDH and pediatric rapid response (RR) events and understanding this impact will help guide future interventions aimed to eliminate health disparities in the inpatient setting.

**Objectives:**

The primary objective of this study is to describe the association between SDH and RR utilization (number of RR events, time to RR event, shift of event and caller). The secondary objective is to determine if SDH can predict hospital length of stay (LOS), ICU transfer, critical deterioration (CD), and mortality.

**Methods:**

A retrospective cohort study was conducted. We reviewed all RR events from 2016 to 2019 at a large, academic, pediatric hospital system including a level 1 trauma center and two satellite community campuses. All hospitalized patients up to age 25 who had a RR event during their index hospitalization were included. Exposure variables included age, gender, race/ethnicity, language, income, insurance status, chronic disease status, and repeat RR event. The primary outcome variables were hospital LOS, ICU transfer, CD, and mortality. The odds of mortality, CD events and ICU transfer were assessed using unadjusted and multivariable logistic regression. Associations with hospital LOS were assessed with unadjusted and multivariable quantile regression.

**Results:**

Four thousand five hundred and sixty-eight RR events occurred from 3,690 unique admissions and 3301 unique patients, and the cohort was reduced to the index admission. The cohort was largely representative of the population served by the hospital system and varied according to race and ethnicity. There was no variation by race/ethnicity in the number of RR events or the shift in which RR events occurred. Attending physicians initiated RR calls more for event for non-Hispanic patients of mixed or other race (31.6% of events), and fellows and residents were more likely to be the callers for Hispanic patients (29.7% of events, *p* = 0.002). Families who are non-English speaking are also less likely to activate the RR system (12% of total RR events, *p* = 0.048). LOS was longest for patients speaking languages other than Spanish or English and CD was more common in patients with government insurance. In adjusted logistic regression, Hispanic patients had 2.5 times the odds of mortality (95% CI: 1.43–4.53, *p* = 0.002) compared with non-Hispanic white patients.

**Conclusion:**

Disparities exist in access to and within the inpatient management of pediatric patients. Our results suggest that interventions to address disparities should focus on Hispanic patients and non-English speaking patients to improve inpatient health equity. More research is needed to understand and address the mortality outcomes in Hispanic children compared to other groups.

## Introduction

The Institute for Healthcare Improvement's “100,000 Lives Campaign” and other patient safety reports contributed to the development of rapid response (RR) systems as an effort to decrease morbidity and mortality associated with healthcare in the United States (1). Studies have demonstrated that RR systems improve hospital outcomes in pediatrics including decreasing rates of cardiopulmonary arrests outside the intensive care unit (ICU), hospital mortality, and critical deterioration events (2–4). In an effort to continue to improve RR systems and patient outcomes, many studies have evaluated several patient and systems factors that influence post-RR outcomes, including elements such as age, medical complexity, underlying diagnosis, primary reason for RR call, occurrence of repeat RR event, time and day of RR, and occurrence of critical deterioration events (5–10).

To our knowledge, no study has evaluated the correlation between social determinants of health, including race, ethnicity, language, or socioeconomic status with RR utilization or post-RR clinical outcomes. Evaluating these associations is imperative to assess the impact of RR systems for several reasons. First, health disparities remain prevalent among racial and ethnic minorities and those who primarily speak a language other than English, and are well-described in inpatient, emergency department, and outpatient settings. In pediatric intensive care units (PICUs) specifically, Latino children, children from families of lower socioeconomic status, or children with public insurance have higher risk of hospital mortality (11–13). Related to language, children admitted to the hospital who have parents with low English proficiency are more than twice as likely to experience an adverse event compared to patients with parents who speak English primarily (14). Secondly, RR events are associated with worse overall patient outcomes compared to patients who do not have RR during their admission. One study demonstrated that patients who had a RR during their admission had 13-times higher odds of mortality compared to patients who did not (7). Critically assessing the association of socio-economic factors with RR systems has potential to guide interventions to eliminate impact of in-hospital disparities on RR utilization and outcomes.

This study aims to fill the gap in current RR system research by describing the association between social determinants of health, specifically race, ethnicity, language, and socioeconomic status, and RR utilization. Our secondary aim is to determine if social determinants of health predict critical deterioration events, hospital length of stay, or mortality after RR events.

## Methods

### Design, Setting, and Population

We conducted a retrospective cohort study of hospitalized children who had RR events from 2016 to 2019 at our large, academic, pediatric hospital system in the southwest United States. Our hospital system serves a diverse, multiethnic metropolis with a population of 57% Caucasian, 45% Hispanic or Latino, 22.6% Black or African American, and 7% Asian, Pacific Islander, and American Indian (15). The hospital system includes a central level 1 trauma campus and two satellite community hospitals. The central campus has 409 general care beds and 132 critical care beds; the satellite campuses have a combined 36 ICU beds and 110 general care beds. Each campus has a 24/7 designated RR team composed of an ICU physician or advanced practice provider (APP), ICU nurse, and ICU Respiratory Therapist who promptly assess, emergently manage, and determine the need for transfer to higher level of care.

We included all hospitalized patients up to age 25 who had RR events, cardiac and/or respiratory arrests from January 2016 to December 2019. All escalation of clinical concerns at our institution, including non-emergent transfers to higher levels of care, occur primarily through the RR process. Patients with RR events during their hospitalization were chosen in order to evaluate the effect of social determinants of health on in-hospital care.

### Data Sources

Data were collected from three sources: a centralized RR database, the electronic medical record (EMR), and the Pediatric Health Information System (PHIS) database, a national database of clinical data from over 49 children's hospitals. PHIS was created to collect administrative and financial data but has since included clinical, treatment, and outcomes-related data, among others (16). We excluded patients with unavailable PHIS data and those who had a RR event outside of our study period. The institutional review board approved this study.

### Exposure Variables

Exposure variables included age, gender, race/ethnicity, language, income, and insurance status. Race and ethnicity were self-identified by parents and were categorized as non-Hispanic White, non-Hispanic Black, non-Hispanic Asian, Hispanic, and non-Hispanic Mixed/Other. Insurance was categorized as commercial, government, and other/self-pay; income was stratified into quartiles. We also included chronic (complex vs. non-complex) and non-chronic disease status as defined by PHIS. Risk of mortality scores were also collected from PHIS to determine risk of mortality scores upon admission as a surrogate for disease severity. Each level retrospectively appraises the mortality risk (minor, moderate, major, and extreme) for each patient on the basis of the patient's age, diagnoses and certain procedures. We identified repeat RR events for the same admission and time from admission to RR. For RR-related variables, the shift in which the RR event occurred, and the primary caller for the event were collected.

### Primary Outcomes

The primary outcome variables were hospital length of stay (LOS), ICU transfer, mortality, and critical deterioration (CD). CD was defined as meeting criteria for initiation of non-invasive positive pressure ventilation, intubation, or vasoactive infusion within 24 h of admission to the intensive care unit. We created a binary yes/no variable for critical deterioration within 24 h of ICU admission. Using the PHIS database, we calculated the total LOS for each admission, and defined mortality as death during the hospitalization in which the RR event occurred.

### Statistical Analysis

Patient and RR characteristics and outcomes were summarized using mean with standard deviation, median with 25th and 75th percentiles, and frequency with percentage. Characteristics and outcomes were compared using *t*-test, Wilcoxon rank sum test, ANOVA, Kruskal–Wallis, Chi-square test, or Fisher's exact test. The odds of mortality, CD events, and ICU transfer were assessed using unadjusted logistic regression and multivariable logistic regression. Length of stay was assessed using unadjusted and multivariable quantile regression. Multivariable regression models included SES characteristics, risk of mortality score, chronic disease status, and time from admission to RR event regardless of statistical significance. Chronic disease status, mortality scores, and time from admission to RR event were included regardless of significance because of a known association with CD events and mortality. Other characteristics were included if statistically significant (*p* < 0.05) in unadjusted regression. Characteristics with more than 5% missing data were not included in the multivariable regression. All analyses are performed using Stata v 15 (StataCorp, College Station, TX).

## Results

**Figure 1:**
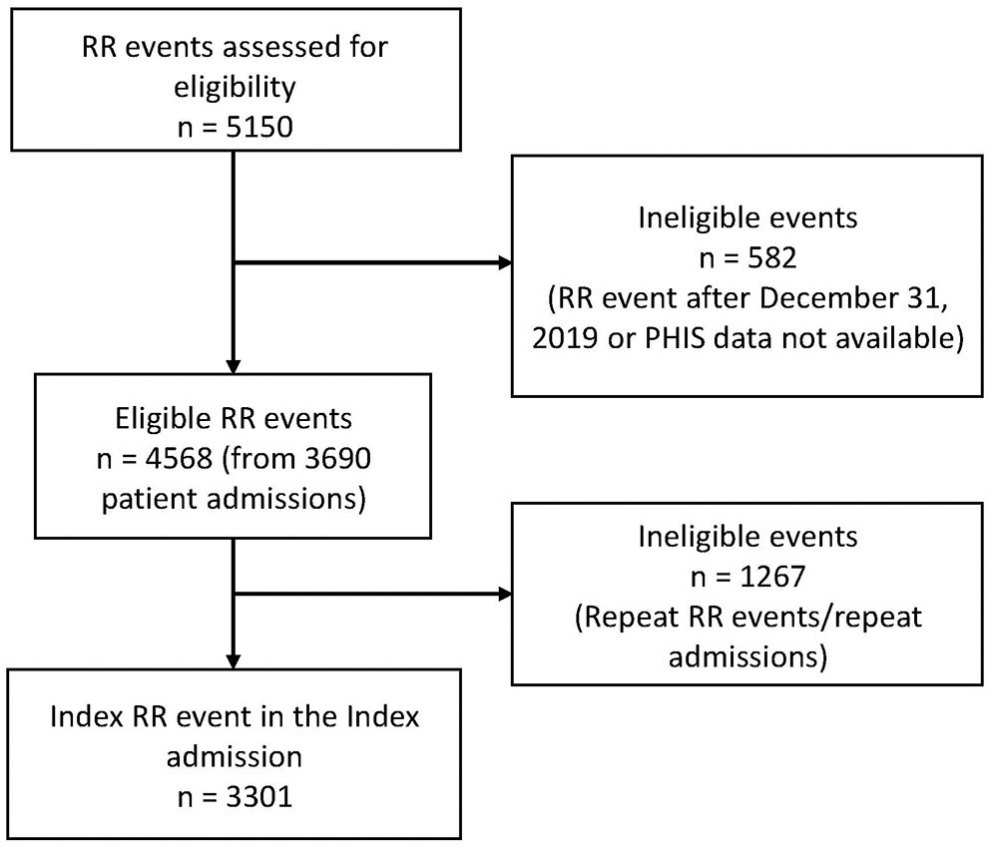
Recruitment flow diagram.

There are a total of 4,568 RR events from 3,690 unique admissions with 83% of patients having only 1 RR event during the admission (range of 1–11 RR events in a single admission). There are 3,301 unique patients with 89% having only one admission in the study time frame (range 1–6 admissions). The cohort was reduced to one RR event per patient using the index RR event from the index admission (Figure 1).

Patient characteristics for the index cohort are displayed in Table 1. Our patient population included patients up to age 25, but only 3.2% (*n* = 104) were over 18 years of age. As expected, preferred language varies according to self-identified race and ethnicity with the highest proportion of Spanish speakers identifying as Hispanic (<0.001). Age, income, and metropolitan housing also varied according to self-identified race and ethnicity. The highest proportion of patients in the lowest income quartile are Hispanic (33.4%), and the highest proportion in the highest income quartile are non-Hispanic Asian (52.1%, *p* < 0.001). The highest proportion of patients with commercial insurance were non-Hispanic white (63.0%) and the highest proportion of government insurance were Hispanic (71%). Patients with complex chronic conditions accounted for 62% of the total number of patients, with the highest proportion among non-Hispanic Asian patients (68.3%, *p* < 0.001). Upon admission, the risk of mortality categories varied by self-identified race and ethnicity (*p* = 0.002).

**Table 1:** Baseline characteristics of patients during index rapid responses in index admission stratified by race/ethnicity.

	**All**	**Non-Hispanic White**	**Non-Hispanic Black**	**Hispanic**	**Non-Hispanic Asian**	**Non-Hispanic Other**	***P*** **-value**
	***N* = 3,301**	***N* = 955**	***N* = 700**	***N* = 1,359**	***N* = 161**	***N* = 58**	
	***N* (%)**	***N* (%)**	***N* (%)**	***N* (%)**	***N* (%)**	***N* (%)**	
**Sex**							
Male	1,806 (55.9)	534 (55.9)	386 (55.1)	768 (56.5)	83 (51.6)	35 (60)	0.726
Female	1,807 (55.9)	421 (44.1)	314 (44.9)	591 (43.5)	78 (48.4)	23 (40)	
**Age**							
<1 year	1,117 (34.6)	336 (35.3)	224 (32.0)	501 (36.9)	44 (27.5)	12 (21)	<0.001
1–3 years	515 (16.0)	145 (15.2)	102 (14.6)	215 (15.9)	36 (22.5)	17 (30)	
4–12 years	862 (26.7)	224 (23.5)	205 (29.3)	365 (26.9)	47 (29.4)	21 (37)	
>12 years	731 (22.7)	248 (26.0)	168 (24.0)	275 (20.3)	33 (20.6)	7 (12)	
**Language**							
English	2,585 (80.0)	921 (96.4)	695 (99.3)	792 (58.3)	121 (75.2)	56 (97)	<0.001
Spanish	569 (17.6)	6 (0.6)	0 (0.0)	563 (41.4)	0 (0.0)	0 (0)	
Other	79 (2.4)	28 (2.9)	5 (0.7)	4 (0.3)	40 (24.8)	2 (3)	
**Insurance**							
Commercial	1,304 (40.3)	602 (63.0)	237 (33.9)	360 (26.5)	84 (52.2)	21 (36)	<0.001
Government	1,823 (56.4)	321 (33.6)	448 (64.0)	965 (71.0)	56 (34.8)	33 (57)	
Other/self-pay	106 (3.3)	32 (3.4)	15 (2.1)	34 (2.5)	21 (13.0)	4 (7)	
**Income quartiles**							
Lowest Quartile−1	763 (24.5)	126 (13.8)	176 (26.0)	442 (33.4)	12 (8.5)	7 (12)	<0.001
2	759 (24.4)	221 (24.2)	144 (21.3)	366 (27.6)	15 (10.6)	13 (23)	
3	760 (24.4)	207 (22.7)	201 (29.7)	295 (22.3)	41 (28.9)	16 (28)	
Highest Quartile−4	832 (26.7)	359 (39.3)	156 (23.0)	222 (16.8)	74 (52.1)	21 (37)	
**Urban category**							
Metropolitan	327 (10.5)	174 (19.1)	48 (7.1)	97 (7.3)	1 (0.7)	7 (12)	<0.001
Non-metropolitan	2,787 (89.5)	739 (80.9)	629 (92.9)	1,228 (92.7)	141 (99.3)	50 (88)	
**Chronic disease status**							
Non-chronic disease	743 (23.0)	213 (22.3)	132 (18.9)	356 (26.2)	28 (17.4)	14 (24)	<0.001
Non-complex chronic disease	478 (14.8)	125 (13.1)	144 (20.6)	181 (13.3)	23 (14.3)	5 (9)	
Complex chronic disease	2,012 (62.2)	617 (64.6)	424 (60.6)	822 (60.5)	110 (68.3)	39 (67)	
**Risk of mortality**							
Minor	1,136 (24.88)	375 (28.13)	246 (26.31)	429 (22.45)	47 (20.52)	19 (25)	0.002
Moderate	933 (20.44)	211 (15.83)	229 (24.49)	406 (21.25)	54 (23.58)	16 (21.05)	
Major	1,135 (24.86)	351 (26.33)	218 (23.32)	461 (24.12)	62 (27.07)	24 (31.58)	
Extreme	1,361 (29.81)	396 (29.71)	242 (25.88)	615 (32.18)	66 (28.82)	17 (22.37)	

Table 2 displays characteristics of RR events according to self-identified race and ethnicity. There was no variation in the number of RR events in the index admission or the shift in which RR events occurred. Though the decision to activate a RR event is often collaborative and multidisciplinary, there were variations among who the documented callers were according to both patient race and ethnicity and patient preferred language. Attendings were more likely to be the caller for the RR event for non-Hispanic patients of mixed or other race (31.6% of events), and fellows and residents were more likely to be the callers for Hispanic patients (29.7% of events, *p* = 0.002). Attending physicians most commonly call for patients who are English speakers (19% of events, *p* = 0.019). Among RR events activated by families, 88% (*n* = 66) were English speakers, 8% (*n* = 6) were Spanish speakers and 4% (*n* = 3) were other language speakers (*p* = 0.048).

**Table 2:** Characteristics of rapid response events stratified by race/ethnicity.

	**All**	**Non-Hispanic White**	**Non-Hispanic Black**	**Hispanic**	**Non-Hispanic Asian**	**Non-Hispanic Other**	***P*** **-value**
	***N* = 3,301**	***N* = 955**	***N* = 700**	***N* = 1,359**	***N* = 161**	***N* = 58**	
	**Median (IQR)**	**Median (IQR)**	**Median (IQR)**	**Median (IQR)**	**Median (IQR)**	**Median (IQR)**	
**Time from admission to RR, hours**	41.2 (17.2, 126.7)	42.8 (17.8, 142.5)	39.5 (16.5,108.4)	37.5 (16.9, 130.0)	59.5 (19.5, 152.4)	44.3 (14.9, 74.6)	0.004
	***N*** **(%)**	***N*** **(%)**	***N*** **(%)**	***N*** **(%)**	***N*** **(%)**	***N*** **(%)**	
**Number of RR event in index admission**							
1	2,697 (83.0)	792 (82.9)	590 (84.3)	1,134 (83.4)	127 (78.9)	54 (93.1)	0.13
2 or more	536 (17.0)	163 (17.1)	110 (15.7)	225 (16.6)	34 (21.1)	4 (6.9)	
**Caller**							
Attending	571 (32.9)	191 (20.3)	123 (17.9)	215 (16.0)	24 (15.1)	18 (31.6)	0.002
Family	73 (4.2)	26 (2.8)	17 (2.5)	21 (1.6)	8 (5.0)	1 (1.8)	
Fellow/resident	865 (49.8)	226 (24.0)	189 (27.4)	400 (29.7)	34 (21.4)	16 (28.1)	
NP/PA	48 (2.8)	16 (1.7)	7 (1.0)	21 (1.6)	4 (2.5)	0 (0.0)	
RN	161 (9.3)	473 (50.3)	348 (50.5)	685 (50.9)	89 (56.0)	21 (36.8)	
RT	18 (1.0)	8 (0.9)	5 (0.7)	4 (0.3)	0 (0.0)	1 (1.8)	
**Day shift**	1,908 (59.1)	547 (57.5)	414 (59.3)	814 (59.9)	97 (60.2)	36 (63.2)	0.759

*RR, rapid response; NP, Nurse Practioner; PA, Physician Assistant; RN, Registered Nurse; RT, Respiratory Therapist*.

Table 3 displays outcomes of RR events. There were no differences in median hospital length of stay (LOS), proportion of patients transferred to the ICU, CD events, or mortality between racial and ethnic groups. LOS was longer in patients who preferred a language other than English (14 days) compared to patients who spoke English or Spanish (9 and 10 days, respectively, *p* = 0.005), and CD events were most common in patients with government insurance (37.6%) compared to those with private insurance (34.2%) or who are self-pay (27.3%, *p* = 0.022). Table 4 displays multivariable logistic regression analyses for outcomes. There were many significant associations that increased odds of mortality even after adjusting for risk of mortality. Age > 1 year is associated with lower odds of ICU transfer and of CD events. Age > 12 years is associated with higher odds of mortality compared to age <1 year (OR = 2.117, *p* = 0.006). Hispanic race was associated with more than two-fold odds of mortality (OR = 2.541, *p* = 0.002), but was not associated with increased odds of ICU transfer (1.043, *p* = 0.737) or CD events (0.979, *p* = 0.838). This association appears to be independent of language, as patients who prefer Spanish did not have increased odds of mortality. Patients with complex chronic diseases also had a significantly higher odds of mortality compared to patients with non-chronic conditions (OR = 8.828, *p* = 0.032).

**Table 3:** Rapid response event outcomes.

	**Transfer to ICU**		**Hospital length of stay, days**		**Critical deterioration events**		**Mortality**	
	***N*** **(%)**	***P*** **-value**	**Median (IQR)**	***P*** **-value**	***N*** **(%)**	***P*** **-value**	***N*** **(%)**	***P*** **-value**
**Race/ethnicity**								
Non-Hispanic White	657 (72.5)	0.075	9 (4, 24)	0.41	331 (34.7)	0.441	24 (2.5)	0.095
Non-Hispanic Black	513 (77.8)		8 (4, 20.5)		253 (36.1)		26 (3.7)	
Hispanic	975 (75.5)		9 (4, 26)		504 (37.1)		64 (4.7)	
Non-Hispanic Asian	107 (69.5)		9 (5, 21)		49 (30.4)		6 (3.7)	
Non-Hispanic Other	42 (76.4)		7 (5, 14)		19 (32.8)		2 (3.4)	
**Language**								
English	1,853 (74.2)	0.098	9 (4, 23)	0.005	945 (35.8)	0.869	97 (3.7)	0.108
Spanish	428 (78.5)		10 (5, 26)		209 (36.5)		25 (4.4)	
Other	65 (73.9)		14 (5, 36)		30 (33.7)		7 (7.9)	
**Insurance**								
Commercial	937 (74.0)	0.597	9 (4, 22)	0.8	457 (34.2)	0.022	54 (4.0)	0.318
Government	1,329 (75.5)		9 (4, 23)		697 (37.6)		68 (3.7)	
Other/Self Pay	80 (76.9)		10 (5, 34)		30 (27.3)		7 (6.4)	

**Table 4:** Multivariable regression for rapid response outcomes.

	ICU transfer^*^			**Hospital length of stay, days^¥^**			Critical deterioration^*^			Mortality^*^		
	**OR**	**95% CI**	***P*** **-value**	**Coefficient**	**95% CI**	***P*** **-value**	**OR**	**95% CI**	***P*** **-value**	**OR**	**95% CI**	***P*** **-value**
**Age**			<0.001			<0.001			<0.001			0.009
<1 year	Reference			Reference			Reference			Reference		
1–3 years	0.76	0.57, 1.01	0.065	−1.424	−2.57, −0.27	0.015	0.68	0.54, 0.86	0.001	0.72	0.29, 1.75	0.472
3–12 years	0.42	0.32, 0.53	<0.001	−2.285	−3.31, −1.26	<0.001	0.53	0.42, 0.65	<0.001	1.48	0.85, 2.57	0.164
>12 years	0.43	0.33, 0.56	<0.001	−1.864	−2.95, −0.77	0.001	0.48	0.38, 0.60	<0.001	2.11	1.24, 3.59	0.006
**Race/ethnicity**			0.107			0.936			0.800			0.026
Non-Hispanic White	Reference			Reference			Reference			Reference		
Non-Hispanic Black	1.40	1.07, 1.82	0.012	0.397	−0.71, 1.51	0.485	1.01	0.80, 1.27	0.886	2.10	1.12, 3.92	0.02
Non-Hispanic Asian	0.99	0.63, 1.56	0.987	−0.375	−2.34, 1.60	0.710	0.78	0.51, 1.19	0.260	1.08	0.34, 3.37	0.891
Non-Hispanic Mixed/Other	1.24	0.62, 2.45	0.532	−0.169	−3.03, 2.70	0.908	0.86	0.47, 1.56	0.622	2.15	0.49, 10.08	0.331
Hispanic	1.04	0.81, 1.33	0.737	0.157	−0.91, 1.22	0.773	0.97	0.78, 1.21	0.848	2.54	1.42, 4.52	0.002
**Insurance**			0.416			0.905			0.430			0.703
Commercial	Reference			Reference			Reference			Reference		
Government	0.88	0.72, 1.08	0.240	−0.188	−1.03, 0.66	0.664	1.04	0.88, 1.24	0.599	0.88	0.57, 1.36	0.572
Other/Self Pay	1.11	0.62, 1.96	0.721	−0.237	−2.66, 2.19	0.848	0.75	0.44, 1.27	0.291	1.28	0.44, 3.66	0.641
**Language**			0.166			0.675			0.862			0.358
English	Reference			Reference			Reference			Reference		
Spanish	1.30	0.99, 1.72	0.059	0.290	−0.87, 1.45	0.625	0.96	0.76, 1.22	0.791	0.73	0.41, 1.28	0.274
Other	0.98	0.51, 1.89	0.963	1.110	−1.76, 3.98	0.448	1.15	0.63, 2.09	0.642	1.68	0.48, 5.84	0.410
**Chronic disease status**			0.994			<0.001			0.001			0.021
Non-chronic disease	Reference			Reference			Reference			Reference		
Non-complex chronic disease	1.01	0.75, 1.36	0.933	0.385	−0.90, 1.67	0.557	0.87	0.67, 1.14	0.321	2.35	0.20, 26.57	0.489
Complex chronic disease	1.01	0.78, 1.31	0.921	2.397	1.30, 3.49	<0.001	0.65	0.52, 0.82	<0.001	8.82	1.20, 64.68	0.032
**Metropolitan**	1.22	0.90, 1.64	0.190	−0.123	−1.43, 1.18	0.853	1.08	0.82, 1.42	0.551	1.10	0.53, 2.26	0.789
**Income quartiles**			0.695			0.909			0.925			0.340
Quartile 1	Reference			Reference			Reference			Reference		
Quartile 2	1.07	0.83, 1.39	0.56	0.047	−1.02, 1.12	0.932	0.98	0.78, 1.22	0.872	0.78	0.44, 1.39	0.406
Quartile 3	0.92	0.71, 1.19	0.540	−0.333	−1.42, 0.76	0.551	0.93	0.74, 1.16	0.545	1.22	0.73, 2.05	0.433
Quartile 4	0.96	0.73, 1.26	0.777	−0.093	−1.23, 1.04	0.873	0.94	0.77, 1.18	0.601	0.79	0.44, 1.44	0.455
**Risk of mortality**			<0.001			<0.001			<0.001			
Minor	Reference			Reference			Reference			Reference		
Moderate	1.63	1.29, 2.07	<0.001	1.497	0.43, 2.55	0.006	2.11	1.68, 2.65	<0.001			
Major	2.90	2.22, 3.78	<0.001	4.845	3.72, 5.96	<0.001	3.18	2.49, 4.04	<0.001	65.01	8.97, 471.21	<0.001
Extreme	4.00	2.99, 5.34	<0.001	9.236	8.06, 10.40	<0.001	4.97	3.86, 6.40	<0.001			

¥ *Hospital LOS coefficients were calculated from multivariable quantile regression*.

* *ICU Transfer, CD and Mortality coefficients were calculated from multivariable logistic regression*.

## Discussion

Our study evaluated the relationship between social determinants of health and RR utilization at a large pediatric hospital system looking at over 4,500 RR events. As demonstrated in Table 1, the demographics are largely representative of the general inpatient population of the study site (15). The effects of race/ethnicity on outcomes have been previously studied in the in-hospital management of pediatric patients (17–22) but this is the first report to focus specifically on the in-hospital RR system.

Similar to other published reports, we noted that Hispanics have increased odds of mortality and adverse events while in the hospital (11, 14, 19). This study adds to the growing body of literature reporting the social disparities in the provision of hospital services (23). The study by Anand et al. (11) demonstrated the effects of a multilevel intervention to reduce the odds of mortality in Latino children. The interventions included among other measures: education regarding cultural competency, recruitment of bilingual staff, and availability of 24-h interpreter services. The cumulative effect of this multi-level intervention strategy eliminated the higher odds of mortality in Hispanic PICU patients than was present in the pre-intervention period. Our hospital system, and others like it can benefit from a similar approach to reduce the disparities that are present within our health care system. Recruiting employees who speak languages other than English, improving access to interpreter services and cultural competency trainings are steps toward providing equitable and quality care for all children. Similarly, the study by Nagarajan et al. (23) showed that minorities had lower satisfaction with the way they experienced care in inpatient pediatrics. Closer examination of the patient and family experience of non-English speakers and Hispanic patients is necessary to determine key processes in which these disparities are perpetuated in the hospital system.

We also noted that attending activation of RR is more common with English speakers. However, it is difficult to draw conclusions from this finding since the decision to call a RR can often reflect a collaborative decision process between the medical and nursing team at the bedside. Although only 17.7% of RRs were activated by attendings, there was no significant difference in the timing of the call (day shift vs. night shift). More studies are needed to evaluate if there is truly more attending communication with English-speakers vs. non-English speakers and to examine the language proficiency of the medical team with non-English speakers.

Notably, our study found that among Spanish and other non-English speakers, families are less likely to activate the RR system suggesting that language barriers impede communication of clinical concerns and awareness of the RR system. This finding is like the study by Chandra et al. (22) which demonstrated that Hispanic children were less likely to be provided with an asthma action plan at discharge, potentially due to a language barrier. Similarly, as demonstrated in studies by Flores and Flower, children who come from households where English is not the primary language experience multiple disparities in medical and oral health, access to care and use of services (24, 25). Our hospital system is already equipped with 24-h interpreter services, though it is unclear from our data how often this resource is utilized for non-English speakers. Additionally, we found that the patients who spoke a language other than English or Spanish had a higher hospital length of stay, though mortality was not significant. Among non-English or Spanish speakers, there is a higher proportion of complex chronic patients. Our hospital system is a large referral center, so this finding somewhat reflects the number of complex patients who come from international settings.

Our study has several limitations, including the use of retrospective databases of a single hospital center (PHIS and our internal RR database). Unfortunately, our data does not demonstrate the severity of illness at the time of the RRT call, although we evaluated critical deterioration events after RR as a surrogate for illness severity in addition to using the risk of mortality score. However, the risk of mortality score was calculated at admission and not at time of RRT. Additionally, our data entry in our internal RR dataset limits the “caller” to one individual and fails to account for the collaborative process between the patient's family, nursing, and medical teams in the activation of an RR. Furthermore, although our study found an increased mortality in patients who had a primary language other than English or Spanish, there is a large confidence interval associated with this finding given the small number of patients included. Additionally, both language and ethnicity are self-reported in our data set and do not fully encompass the diversity represented in this large category. While our findings showed differences related to race and ethnicity, we acknowledge that these are constructs that may not fully grasp the complexity of social and environmental influence on health.

## Conclusion

Our study adds to the growing body of literature that demonstrates that health care inequities exist not only in accessing health care, but also within the inpatient utilization of response service and outcomes of pediatric patients. Based on our results, we suggest that patients who have primary languages that are not English be recognized as a high-risk population, with future efforts directed to improving communication and enhancing cultural competency in order to reduce their in-hospital risk.

## Data Availability Statement

The original contributions presented in the study are included in the article/supplementary material, further inquiries can be directed to the corresponding author.

## Ethics Statement

The studies involving human participants were reviewed and approved by Baylor College of Medicine. Written informed consent from the participants' legal guardian/next of kin was not required to participate in this study in accordance with the national legislation and the institutional requirements.

## Author Contributions

NL, DA, JB, AB, and NA: conceptualization. NL, DA, DG, AB, and NA: methodology. NL, DA, JB, DG, and NA: investigation. DG: data curation. NL, DA, AB, PC, and NA: writing. All authors have read and agreed to the published version of this manuscript.

## Funding

Publication costs were generously supported by the Texas Children's Hospital Young Investigators Endowed Fund.

## Conflict of Interest

The authors declare that the research was conducted in the absence of any commercial or financial relationships that could be construed as a potential conflict of interest.

## Publisher's Note

All claims expressed in this article are solely those of the authors and do not necessarily represent those of their affiliated organizations, or those of the publisher, the editors and the reviewers. Any product that may be evaluated in this article, or claim that may be made by its manufacturer, is not guaranteed or endorsed by the publisher.
